# An Introduction to the Study of Clinical Medicine

**Published:** 1874-03

**Authors:** 


*An Introduction to the Study of Clinical Medicine: Being a Guide to the investigation of Disease for the Use of Students.* By Octavius Sturges, M. D., Cantab. Philadelphia: Henry C. Lea, 1873. Buffalo: T. Butler & Son.

This little book is intended to be a guide to students in the study of medicine at the bedside. It teaches the student, who is supposed to know what he wishes to see, how to see it. The author encourages the student to trust to his own powers of observation and to cultivate them. As a guide to the proper study of clinical medicine we think it will be well received.

